# Qatar Prediction Rule Using ED Indicators of COVID-19 at Triage

**DOI:** 10.5339/qmj.2021.18

**Published:** 2021-08-11

**Authors:** Sameer A. Pathan, Caroline E. Thomas, Zain A. Bhutta, Isma Qureshi, Sarah A. Thomas, Jibin Moinudheen, Stephen H. Thomas

**Affiliations:** ^1^Hamad Medical Corporation, Doha, Qatar E-mail: IQureshi@hamad.qa; ^2^Al Wakrah Hospital, Hamad Medical Corporation, Doha, Qatar; ^3^School of Public Health and Preventive Medicine, Monash University, Melbourne, Australia; ^4^Southbank International School, Westminster, London, UK; ^5^Bachelor Candidate in Medical Biosciences, Faculty of Medicine, Imperial College London, UK; ^6^Blizard Institute of Barts & The London School of Medicine, Queen Mary Univ. of London, UK

**Keywords:** COVID-19, COVID positive, triage, predict, emergency department, Qatar

## Abstract

Introduction: The presence of Severe Acute Respiratory Syndrome Coronavirus-2 (SARS-CoV-2) and its associated disease, COVID-19 has had an enormous impact on the operations of the emergency department (ED), particularly the triage area. The aim of the study was to derive and validate a prediction rule that would be applicable to Qatar’s adult ED population to predict COVID-19-positive patients.

Methods: This is a retrospective study including adult patients. The data were obtained from the electronic medical records (EMR) of the Hamad Medical Corporation (HMC) for three EDs. Data from the Hamad General Hospital ED were used to derive and internally validate a prediction rule (Q-PREDICT). The Al Wakra Hospital ED and Al Khor Hospital ED data formed an external validation set consisting of the same time frame. The variables in the model included the weekly ED COVID-19-positivity rate and the following patient characteristics: region (nationality), age, acuity, cough, fever, tachypnea, hypoxemia, and hypotension. All statistical analyses were executed with Stata 16.1 (Stata Corp). The study team obtained appropriate institutional approval.

Results: The study included 45,663 adult patients who were tested for COVID-19. Out of these, 47% (n = 21461) were COVID-19 positive. The derivation-set model had very good discrimination (c = 0.855, 95% Confidence intervals (CI) 0.847–0.861). Cross-validation of the model demonstrated that the validation-set model (c = 0.857, 95% CI 0.849–0.863) retained high discrimination.

A high Q-PREDICT score ( ≥ 13) is associated with a nearly 6-fold increase in the likelihood of being COVID-19 positive (likelihood ratio 5.9, 95% CI 5.6–6.2), with a sensitivity of 84.7% (95% CI, 84.0%–85.4%). A low Q-PREDICT ( ≤ 6) is associated with a nearly 20-fold increase in the likelihood of being COVID-19 negative (likelihood ratio 19.3, 95% CI 16.7–22.1), with a specificity of 98.7% (95% CI 98.5%–98.9%).

Conclusion: The Q-PREDICT is a simple scoring system based on information readily collected from patients at the front desk of the ED and helps to predict COVID-19 status at triage. The scoring system performed well in the internal and external validation on datasets obtained from the state of Qatar.

## Background & Study Aims

The worldwide persistence of Severe Acute Respiratory Syndrome Coronavirus-2 (SARS-CoV-2) and its associated disease, COVID-19 has had a significant impact on emergency department (ED) operations planning. One arena that has been affected is triage, where, based upon limited initial information, decisions must be made regarding patient streaming. Ideally, all patients presenting at triage should be isolated and treated as if they are COVID-19 positive. In some situations, particularly in the event of a COVID-19 resurgence, resource restrictions may preclude such a conservative approach.

In a potential COVID-19 resurgence, triage according to COVID-19 risk stratification could be helpful to inform decisions regarding the allocation of ED resources (*e.g.,* isolation room versus waiting room with a mask) rather than waiting for results from imaging or laboratory testing.^1^ In order to ascertain whether triage data could form a basis for elucidation of a useful prediction tool, the investigators designed the *Qatar Prediction Rule using ED Indicators of COVID-19 positive at triage* (Q-PREDICT).

The study aims were to derive and validate a prediction rule that would be applicable to Qatar’s adult ED population. The secondary aim was to outline a mixed-method approach to generating a prediction rule that could be useful in other countries.

## Methods

### Design & setting

This was an ED-based electronic medical record (EMR) study of routinely collected data. Analysis and modeling were executed using EMR database information as well as publicly available nationwide COVID-19 information disseminated by Qatar’s Ministry of Public Health.

The Hamad General Hospital emergency department (HGH-ED) is one of the largest tertiary care centers of all the hospitals in the Hamad Medical Corporation (HMC). It has an annual patient inflow of approximately 400,000 cases. The Al Wakra Hospital (AWH) ED is located approximately 10 miles south of Doha and has an annual patient intake of 210,000. The Al Khor Hospital (AKH) ED, located approximately 30 miles north of Doha, has an annual intake of 160,000 patients. The HMC ED’s Polymerase chain reaction (PCR) testing is performed at the HMC virology lab, where SARS-CoV-2 testing is executed using real-time PCR for nasopharyngeal and oropharyngeal swabs. For the study purpose, case definition of “COVID-19 positive” was based on real-time PCR testing. The turnaround time for the HMC PCR is approximately six hours.

### Patient inclusion criteria and data selection

Adult patients (age >17 years) visiting the ED between March 7 and July 31, 2020 were included in the study. Data were analyzed as reported by the EMR. All data definitions and categorization decisions were made *a priori*. The various data categories are outlined in Appendix 1 (Section 1 (c)).

Demographic data included age, nationality, mode of arrival in the emergency department, and triage acuity assigned using the Canadian Triage and acuity scale. Vital signs consisting of systolic blood pressure, respiratory rate, pulse oximetry (Pox), and temperature were dichotomized.

### Data handling and analysis

The data were imported from the system’s EMR database into a spreadsheet (Excel, Microsoft Corporation, Redmond, WA). Data were then imported into statistical software for analysis and plotting. All statistical analysis was executed with Stata 16.1 (StataCorp, College Station, TX). The level of significance was set at *p* = 0.05. Confidence intervals (CIs) were calculated at the 95% level. Further details on descriptive and univariate analyses and details on generating a logistic regression model using predictors identified by random forest are in Appendix 1 (Section 1 (d)).

Due to the relatively frequent occurrence of the outcome of interest (COVID-19 positivity), the logistic regression “rare-disease assumption” was not met; thus, the focus of regression was statistical significance, rather than estimation of odds ratios (ORs) and effect sizes.^2,3^

### Generating a logistic regression model using predictors identified by random forest

To assess the dichotomous endpoint of COVID-19 positivity (the dependent variable), multivariate logistic regression was used to simultaneously adjust for multiple covariates. In order to avoid overfitting and also to optimally consider nonlinearities and interactions, ensemble learning (random forest) statistical techniques were used for the initial selection of variables to assess by logistic regression.^4–7,8^ Details on the random forest methodology used in Q-PREDICT, including assessment of error convergence and hyperparameter selection, are provided in Appendix 1 (Section 1 (d)).

The initial multivariate regression model was developed on a derivation set of HGH data (one-half of the overall HGH dataset, randomly selected). Post-estimation model assessment included assessments of discrimination (*c* statistic) and calibration (Brier score and Spiegelhalter’s *z*).^8,9^ the *c* statistic, equal to the area under the receiver operator characteristic (ROC) curve, was used as an important, if imperfect, indicator of predictive model utility in balancing sensitivity and specificity.^10,11^

Since the logistic regression model was developed on a derivation set of half of the HGH data, the next step was to apply the model to the other half of the HGH data (the internal validation set).

### Generation Of The Q-predict Rule

After derivation and validation of the logistic regression model, the next step was to translate the variables into a score. When modeling assumptions are met, the results from a logistic regression model (or any generalized linear model) can be used to generate a given case’s probability: sum the products of each variable’s value and its coefficient (parameter), and add an error term. In this study, straightforward translation of the logistic regression model was not applicable due to violation of the rare-disease assumption. The overall COVID-19 positivity rate was well beyond the traditional 10% level above which the odds ratio overestimates relative risk.^3^ Straightforward computations to change OR to relative risk (RR) have been suggested,^12^ but they are potentially biased and are not fit to generate reliably accurate relative risk measures in a confounded dataset.^13^

Given the limitations of OR with the high rate of COVID-19 positivity, the ORs from logistic regression were used to generate a prediction score based on those ORs’ general rankings (not their precise values). As an arbitrary starting point, the variables with higher ORs were set to generate two points toward the final score; variables with lower ORs contributed one point.

### Internal and external validation of Q-PREDICT

Just as the logistic regression model was generated on the HGH derivation set and internally validated on the HGH validation set, the first step in assessing the Q-PREDICT rule was to evaluate its performance on HGH data. To avoid favorably biasing the rule performance by mixing derivation and validation data, Q-PREDICT was first evaluated on the derivation set and then on the validation set.

The most important function of Q-PREDICT is to assist in triage (literally, “to sort”). Since the very function of triage is to discriminate (*i.e.,* more likely from less likely cases), the most important calculation regarding Q-PREDICT was its discrimination capability (*c*).

As recommended by Steyerberg *et al.*, “any performance measure should be corrected for optimism.“^10^ The *c* statistic was therefore calculated with use of a *k-*fold cross-validation technique, which allowed for the generation of a 95% CI for *c*.^14^ Additional measures that were also calculated at varying Q-PREDICT cutoffs included sensitivity, specificity, and positive and negative predictive values (PPV and NPV).

After Q-PREDICT was derived from the HGH derivation set, and the above performance parameters were calculated for those data, the same measures were calculated for the HGH internal validation set. Finally, the Q-PREDICT performance measures were calculated for the external validation set which consisted of COVID-19-positive patients included in the study during the study period in Qatar.

## Results

### Characteristics of the overall study population

The study included a total of 45,663 ED adult patients in whom COVID-19 testing was performed Table 1. Of the total group, 24,923 were seen at HGH during the 147-day, 21-week study period of March 7, 2020 through July 31, 2020. AKH and AWH EDs accounted for 5,565 and 15,175 COVID-19 tests, respectively.

**Figure 1.**
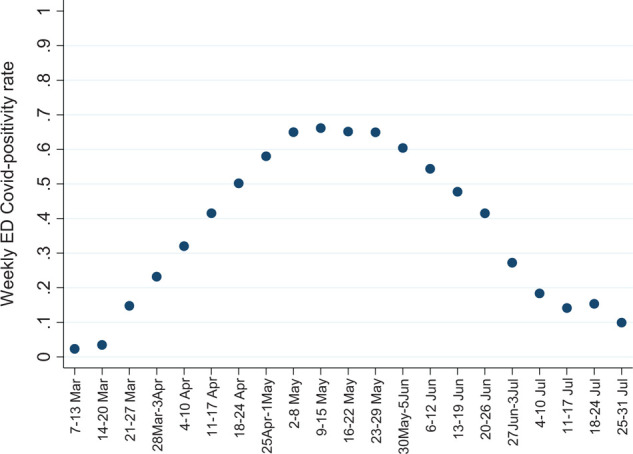
Weekly COVID-19 positivity rates in emergency departments of three Qatari hospitals

The overall COVID-19 positivity rate, assessed across the entire study duration, was 21,461 of 45,663 (47.0%). Weekly ED COVID-19 case numbers, as well as weekly proportions across centers, were included in the study Figure 1. The COVID-19 positive rates are shown in Appendix 1 (Section 1 (b)). For ED centers included in the study, COVID-19 testing over the study duration resulted in hospital-specific COVID-19 positivity rates of 50.0% for AKH, 50.8% for AWH, and 44.0% for HGH; the lower proportion of COVID-19-positive cases at HGH, as compared with the other sites was statistically significant (*p* < 0.001).

### Detailed patient characteristics from the three study sites

Initial vital signs and laboratory findings of the HGH, AKH, and AWH cases are shown in Table 2. The vital sign information is presented in more detail than was utilized for the modeling (which, for vital signs other than temperature, was based on dichotomized categories). The intent of this detailed presentation is to convey more information about the sites’ patient populations and also the existence of missing data (the proportions of which were specified for any variable with at least 0.2% missing data at any site). The vital sign categories as presented here are those that were used to assess whether polytomous categorization of vital signs would improve the prediction result.

The disposition information from each study site is shown in Table 3. As in the study center, the term “eloped” is used to describe patients who were seen by a physician but left the ED in the absence of a discharge order.

### Evaluation of the logistic regression model

The derivation-set model had very good discrimination (*c* = 0.855, 95% CI 0.847–0.861). Post-estimation model assessment was consistent with acceptable calibration (Spiegelhalter’s *z* test *p* = 0.780). Cross-validation of the model on the HGH internal validation set is detailed in Appendix 2 (Section 2 (d)); analysis demonstrated that the validation-set model (*c* = 0.857, 95% CI 0.849-0.863) retained the validation dataset’s high level of discrimination Table 4.

**Figure 2.**
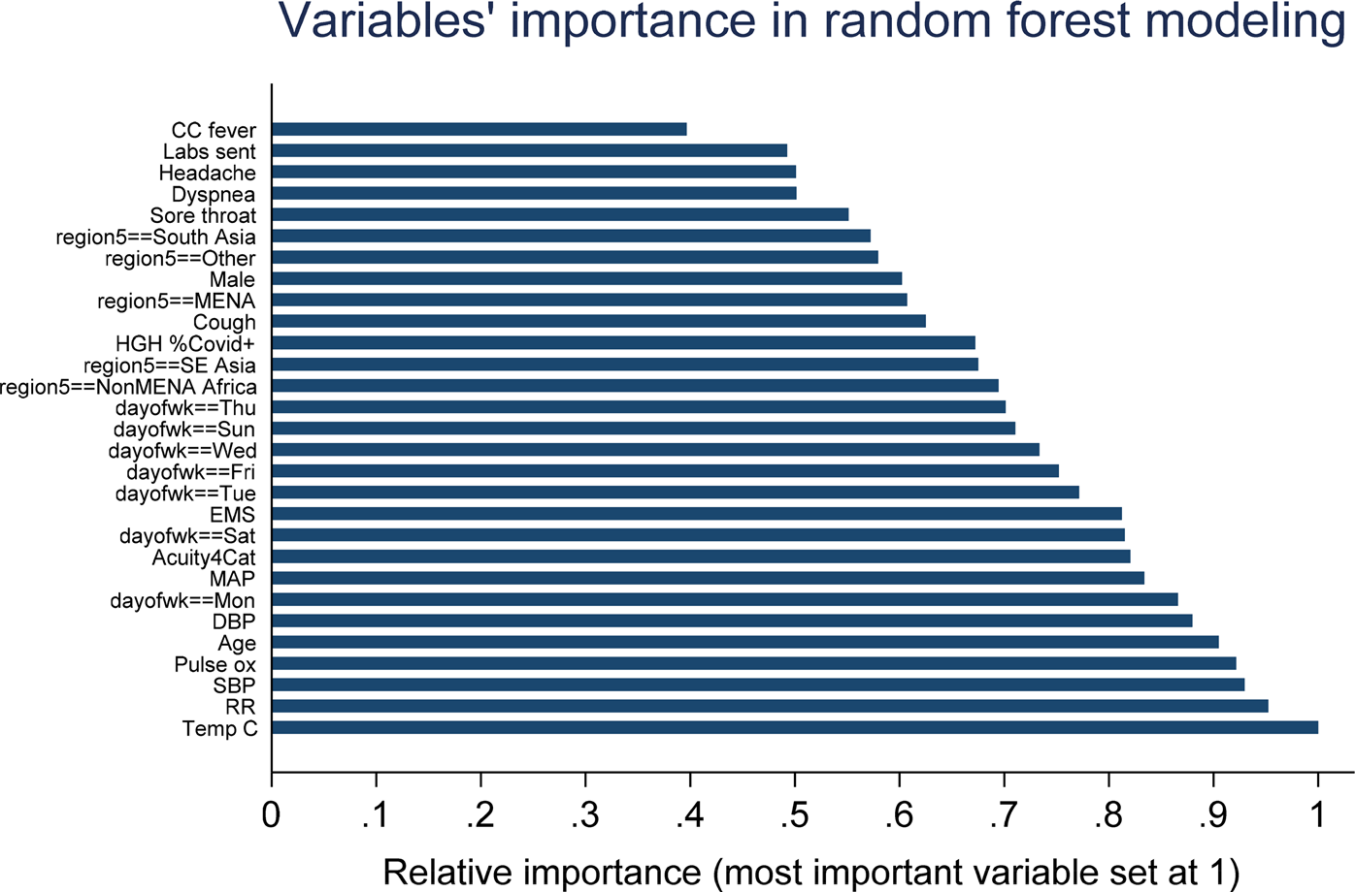
Relative importance plot of Q-PREDICT candidate predictors

After execution of the random forest, a plot of the important variables was generated (Figure 2). The plot was used to exclude variables (*e.g.,* chief complaint of fever, which scored the lowest) from further exploration as contributors to the prediction rule.

### Generating the prediction rule

The results of logistic regression were used to assign point scores to the variables of interest, as described in Appendix 2 (Section 2 (e)). The resulting Q-PREDICT rule is shown in Table 3.

### Performance Of The Q-predict Rule

**Figure 3.**
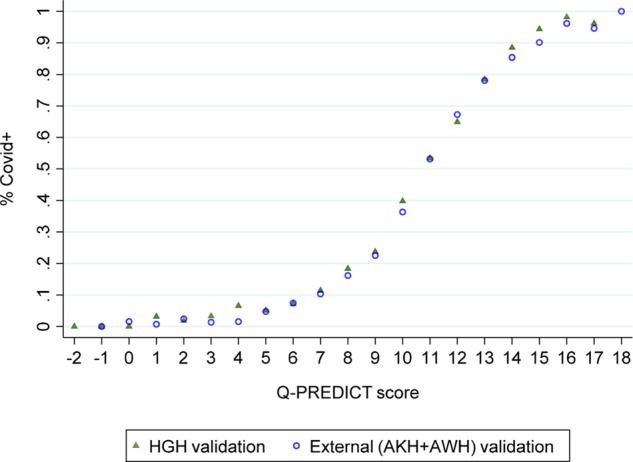
Q-PREDICT score and COVID+ probability (internal and external validation sets)

**Figure 4.**
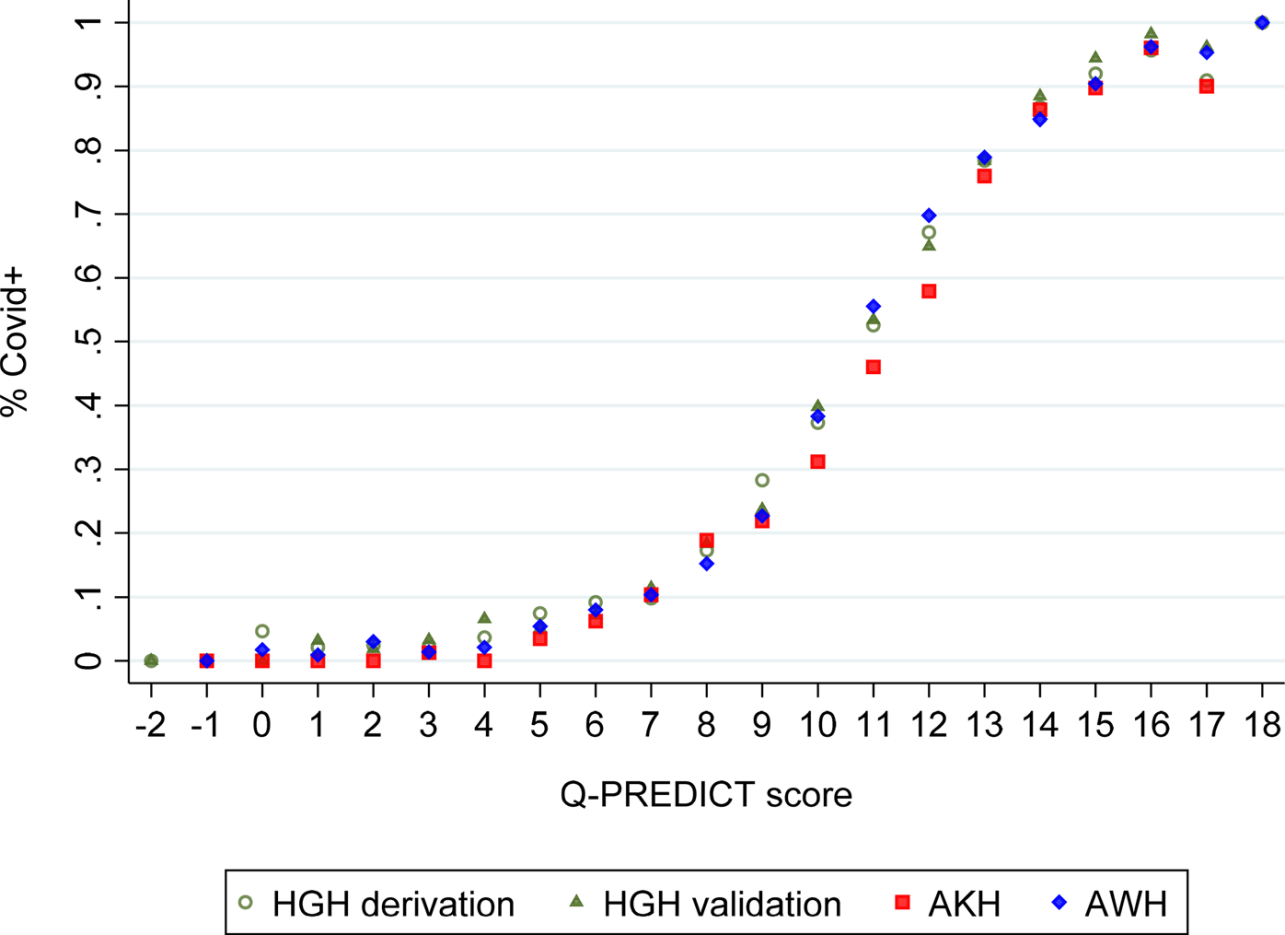
Q-PREDICT performance: HGH (derivation and validation sets), AKH, and AWH

The Q-PREDICT score was generated for the 43,478 cases (95.2% of 45,663) with complete data allowing score calculation. The rule was tested first on the HGH validation dataset and then on the external validation dataset (AKH + AWH). The Q-PREDICT score’s sigmoidal shape is associated with a rising Q-PREDICT and an increasing likelihood of COVID-19 positivity in the internal and external validation data (Figure 3). Appendix 2 (Figure 4 (A9)) reports the score performance of each of the hospital-site datasets. Figure 4 shows the Q-PREDICT performance HGH (derivation and validation sets); AKH and AWH.

**Figure 5.**
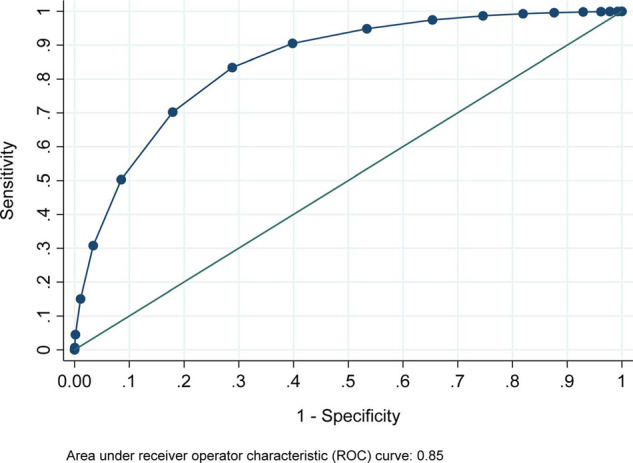
Sensitivity and specificity for Q-PREDICT in internal and external validation data

The ROC curve for the combined group of HGH internal validation data and AKH + AWH external validation data is shown (Figure 5). For the HGH internal validation set, the *c* (area under ROC) was 0.85 with cross-validated 95% CI 0.84–0.85. For the external validation data, *c* was 0.84 with 95% CI 0.83–0.85. For the combined data from internal and external validation *c* was 0.85 with 95% CI 0.84–0.85.

Since disease prevalence is incorporated into its calculation, Q-PREDICT is poorly suited to calculations of PPV (which depend on prevalence). With that limitation, illustrative calculations were made in the combined internal and external validation data using the *a posteriori*-defined ranges of < 7 to indicate low risk and >12 to indicate high risk.

As compared with lower Q-PREDICT ( < 13), a high Q-PREDICT (>12) is associated with a nearly 6-fold increase in the likelihood of COVID-19 (likelihood ratio 5.9, 95% CI 5.6–6.2); 84.7% (95% CI, 84.0%–85.4%) of those with a high score have COVID-19. There is a corresponding high specificity (*i.e.,* absence of high score in COVID-19-negative cases) of 91.5% (95% CI, 91.0%–91.9%).

As compared with a Q-PREDICT >6, a low Q-PREDICT ≤ 6 is associated with a nearly 20-fold increase in the likelihood of COVID-19 negativity (likelihood ratio 19.3, 95% CI 16.7–22.1); 95.4% (95% CI, 94.7%–96.0%). There is a corresponding high specificity (*i.e.,* absence of a low score in COVID-19-positive cases) of 98.7% (95% CI 98.5%–98.9%).

## Discussion

With no proven specific treatment (except possibly dexamethasone for severe disease)^15^ and no vaccine as of December 2020, In the event of a COVID-19 resurgence in Qatar, it is possible that resource demands could outstrip availability. For these and other related reasons, there is potential ED operations utility in developing a stratification method to discriminate between those with higher and lower COVID-19 risk. A desire to develop the ability to separate triage patients with higher versus lower COVID-19 risk led to the development of Q-PREDICT. Pending the development of rapid testing for COVID-19, screening for the disease requires a wait time of many hours (at least six) for PCR results. While rapid techniques such as loop-mediated isothermal amplification (LAMP) or rapid IgG/IgM testing may prove useful, such tests tend to suffer from a combination of unreliability and unavailability.^1,16,17^ the study’s aim was to identify, in ED patients who were undergoing COVID-19 testing, triage-assessable factors that could be used to stratify the risk of positive COVID-19 results. Therefore, Q-PREDICT aimed to formulate a simple scoring approach that used only information that was readily available at triage in EDs in Qatar.

The focus on triage-available information is both a strength and a weakness of the Q-PREDICT approach. There are limitations associated with not considering laboratory testing. Abnormalities such as hyponatremia are well-known to be associated with the presence and severity of COVID-19.^18–20^ Potentially related to the syndrome of inappropriate antidiuretic hormone section, sodium levels may be particularly useful as COVID-19 indicators in patients with atypical, nonrespiratory presentations.^21,22^ A COVID-19 risk stratification system could benefit from the incorporation of information related to white blood cell (WBC) count. Either WBC or the differentials appear to have utility in COVID-19 diagnosis and/or prognosis.^19,23–29^ Other laboratory data of suggested utility in COVID-19 diagnosis or risk stratification include glucose^20^ and bicarbonate.^27,30,31^ Analysis of patients with radiographic COVID-19 pneumonia identified normal presentation WBC in 90% of cases.^32^

On balance, Q-PREDICT’s limitation to triage obtainable information does not represent a critical weakness. Instead, the score development’s concentration on information routinely obtained at triage translates into immediate results.

Information routinely obtained at triage includes vital signs, chief complaints, and the patient’s region of origin. Some of these data are well-known to be associated with COVID-19 risk or severity. Hypertension, for example, is a known risk factor for more severe COVID-19.^29,33^ Chief complaints such as cough, fever, dyspnea, and myalgia are commonly reported in patients with laboratory-confirmed COVID-19.^34–36^

The nonspecificity of the various chief complaints and vital signs findings renders these data unfit for application as individual drivers of risk stratification. Furthermore, any calculations of associations found in a retrospective dataset analysis would incur substantial risk of overfitting of the data. Simple modeling using these data would likely produce a model that performed very well at describing associations in the study dataset, without performing well to prospectively stratify COVID-19 risk in a different patient group.

The modeling step of Q-PREDICT was necessary given the “black-box” nature of random forest algorithm generation.^37,38^ Ensemble learning techniques (such as random forest) are less interpretable than more traditional (and more easily interpreted) approaches such as logistic regression.^38^ The rule extraction process used in Q-PREDICT took the variables identified by random forest methods and generated a simple logistic regression model that had excellent performance in the derivation and in both internal and external validation data. While the performance of Q-PREDICT was more important than the details of rule development, the current study has limitations. One limitation was the fallibility of the PCR testing itself. An editorial from the American Society for Microbiology concluded that the true sensitivity of PCR testing is unknown, but certainly not 100%.^39^ Additional study limitations emphasize the unproven generalizability of results outside of Qatar. The Q-PREDICT, derived from HGH data and validated both on HGH and other national data, is demonstrably accurate nationwide in Qatar. However, each country’s national trends and risk factors differ. The key messages of this study lie in both the methods and the final product of the COVID-19 risk stratification tool. While the score itself may or may not be found applicable in other countries, the steps taken to generate the score are quite portable. Q-PREDICT employed a methodology not readily found elsewhere in score generation. The use of random forest techniques excluded relatively noncontributory variables and reduced overfitting. Logistic regression then provided relative risk estimates that were translated into weighting of variable scores. The Q-PREDICT score can be easily regenerated by incorporating the method given in the report and the appendices.

### Conclusion

The Q-PREDICT is a simple scoring system helps in predicting COVID-19 status at triage from the information readily available at the front desk of an emergency department. Q-PREDICT performed well in the internal and external validation on datasets obtained from the state of Qatar.

### Author Contributions

SP: Formulation of the research question. Formulation of the statement of hypothesis and drafting of the initial manuscript.

CT: Formulation of the statement of hypothesis and with the manuscript.

ZB: Data collection and analysis. Manuscript review.

IQ: Data collection and analysis. Manuscript review.

SAT: Data setup and analyses. Generation of graphs and tables.

JM: provided research data.

SHT: Formation of the concept and design of the manuscript. Data analysis and interpretation. Final approval of the manuscript for submission for publication.

### Funding

None

### Conflict of Interest

None for all authors

## Figures and Tables

**Table 1:** Characteristics of ED patients (n = 45,663) undergoing COVID testing

Variable (*n* with available data; % of 45,663)	n or %

Age (n = 45,626; 99.9%), years	

Median (interquartile range)	36 (29–46)

Younger adult ( < 40)	60% (n = 27375)

Older adult (40+)	40% (n = 18251)

Male (n = 45,647; 99.96%)	79.6% (n = 36335)

Region of nationality (n = 45,663; 100%)	

South Asia	61.1% (n = 27900)

Middle East & North Africa (MENA)	25.3% (n = 11553)

Southeast Asia	7.3% (n = 3333)

NonMENA Africa	5.0% (n = 2283)

Other	1.3%

	(n = 594)

Arrival by ambulance (n = 45,663; 100%)	35.1%

	(n = 16028)

Triage acuity (n = 45,250; 99.1%)	

Critical or arrest	7.0% (n = 3168)

Intermediate	35.3% (n = 15973)

Low or lowest	57.7%

	(n = 26109)

Chief complaints (n = 45,663; 100%)	

Fever (reported by patient)	54.4% (n = 24841)

Cough	22.7% (n = 10365)

Sore throat	9.5%

	(n = 4338)

Shortness of breath	6.0% (n = 2740)

Headache	5.2% (n = 2374)


**Table 2:** Initial findings and laboratory results

Variable	AKH (n = 5,565)	AWH (n = 15,175)	HGH (n = 24,923)

Triage temperature			

< 37.0°C	25.5%	36.1%	32.6%

37.0°C–37.9°C	36.6%	39.4%	41.5%

38.0°C–38.9°C	26.3%	17.8%	18.5%

39°C or higher	11.3%	4.2%	6.5%

Temperature unrecorded	0.4%	2.5%	0.9%

Triage respiratory rate			

< 12	0.1%	0.1%	0.1%

12–19	51.4%	73.1%	59.2%

20 or higher	47.8%	21.5%	38.5%

Respiratory rate unrecorded	0.7%	5.3%	2.2%

Pulse oximetry saturation at triage			

< 90%	0.5%	0.8%	1.0%

90%–94%	1.3%	2.2%	3.6%

95%–100%	97.8%	94.4%	94.3%

Pulse oximetry unrecorded	0.3%	2.6%	1.2%

Blood pressure (BP) at triage			

Hypotensive	0.4%	0.3%	0.8%

Normal BP	61.6%	65.9%	65.9%

Hypertensive	37.4%	31.4%	32.0%

BP unrecorded	0.5%	2.3%	1.3%

Any laboratory tests ordered	48.7%	32.0%	59.0%


**Table 3:** Information about the disposition from each study site.

Variable	AKH (n = 5,565)	AWH (n = 15,175)	HGH (n = 24,923)

Died in the emergency department (ED)	0%	.04%	0.4%

Admitted to any location	23.2%	24.0%	41.3%

Discharged from the ED	74.7%	73.1%	56.8%

Left the ED against advice or eloped	1.8%	1.2%	1.1%

ED disposition not recorded	0.4%	1.7%	0.4%


**Table 4:** Logistic regression model developed from the HGH derivation set.

Predictor variable for outcome of ED COVID-19-positivity^*^	Odds ratio (OR)	95% CI for OR

Weekly ED COVID-19-positivity rate	1.05	1.048–1.053

Age: Older adult (>40) *vs.* younger adult	1.40	1.27–1.54

Region of nationality		

South Asia *vs.* other nationalities	5.65	5.06–6.32

Southeast Asia *vs.* other nationalities	2.70	2.26–3.23

Africa, not Middle East North Africa (MENA) *vs.* other	2.42	1.96–2.98

Triage acuity (increasing score corresponds to lower acuity)	1.52	1.40–1.65

Chief complaint includes cough	1.67	1.49–1.88

Vital signs		

Systolic blood pressure either normal or high (*vs.* low)	3.39	1.63–7.05

Tachypnea (respiratory rate at least 20)	1.22	1.11–1.35

Fever (temperature at least 38°C)	5.32	4.77–5.94

Hypoxemia (pulse oximetry < 95%)	1.86	1.48–2.35

